# Clinical practice on treatment of primary traumatic anterior shoulder dislocation: a multidisciplinary guideline

**DOI:** 10.2340/17453674.2026.46434

**Published:** 2026-08-10

**Authors:** Marianne L VAN GASTEL, Karin M C HEKMAN, Femke BOON, Stijn D NELEN, Niels W L SCHEP, Olivier A J VAN DER MEIJDEN, David N BADEN, M A Thomas JONKERGOUW, Louise E HUYGEN, Astrid C J BALEMANS, Mitchel GRIEKSPOOR, Robert Jan DERKSEN, Michel P J VAN DEN BEKEROM

**Affiliations:** 1 Department of Orthopedic Surgery, Ziekenhuis Amstelland, Amsterdam; 2 Department of Surgery, Zaandam Medical Center, Zaandam; 3 IBC-Amstelland, Amstelveen; 4 Department of Surgery, Radboud University Medical Center, Nijmegen; 5 Department of Hand and Wrist Surgery, Maasstad Ziekenhuis, Rotterdam; 6 Department of Orthopedic Surgery, Albert Schweitzer Ziekenhuis, Dordrecht; 7 Emergency Department, Diakonessenhuis, Utrecht; 8 Dutch Patient Federation, Utrecht; 9 Department of Radiology, Sint Maartenskliniek, Ubbergen; 10 Knowledge Institute of the Dutch Association of Medical Specialists, Utrecht; 11 Department of Orthopedic Surgery, Shoulder and Elbow Unit, OLVG, Amsterdam; 12 Department of Human Movement Sciences, Vrije Universiteit Amsterdam, The Netherlands

## Abstract

**Background and purpose:**

Primary traumatic anterior shoulder dislocations are common and can have long-term personal implications for quality of life and the use of the arm for occupational activities and sport participation. A patient care pathway has been reported to contribute to improved quality of care and a reduction in re-dislocations. We developed clinical practice guidelines intended to provide healthcare professionals with an updated pathway for the optimal diagnosis and management of traumatic primary anterior shoulder dislocations.

**Methods:**

The clinical practice guidelines were developed by a multidisciplinary committee for the following topics: (i) diagnostic evaluation, (ii) reduction technique, (iii) pain management during reduction, (iv) immobilization, (v) physical therapy, (vi) risk factors for recurrent dislocation, and (vii) primary surgical shoulder stabilization.

**Results:**

On admission, dislocations require prompt treatment after diagnostic imaging. The choice of reduction technique is based on individual experience, favoring a biomechanical approach without analgesics. If reduction fails, procedural sedation and analgesia are recommended. To confirm reduction and rule out complications, imaging is repeated, and 1 week of relative immobilization is initiated. Following the acute phase, physical therapy targets early restoration of function, coordination, and proprioception. Risk factors of recurrence include young age and male sex, and surgical intervention may be discussed in the case of age < 40 years, contact athletes, and significant bone loss. The recommendations given are mostly based on data of low GRADE evidence, supplemented with expert opinion.

**Conclusion:**

These guidelines emphasize the importance of timely, efficient, and safe management in the emergency department. Pre- and post-reduction diagnostics are crucial for safe reduction and appropriate management. In the case of complications or recurrent instability, timely management enhances long-term outcomes.

A primary traumatic anterior shoulder dislocation has an incidence between 12 and 26 per 100,000 person-years [1,2]. Dislocations frequently result from direct blows and falls. In males, dislocations predominantly occur during sports participation or recreational activities, whereas in females, they often occur at home. Acute dislocations are typically reduced in the emergency department and should, following an initial assessment that excludes any associated fractures, be addressed promptly upon arrival [3,4]. Prompt reduction is essential to mitigate the effects of compression of the neurovascular structures, tissue stretch, and muscle spasm tending to increase with time to reduction [5].

Independent of treatment choice, primary shoulder dislocations can have long-term implications for quality of life, occupation, and sports [6]. Additionally, the societal costs are substantial, with an estimated mean of €6,914, €5,284, and €4,061 for the 1st, 2nd, and 3rd nonoperatively treated dislocations, respectively [7].

The previously published “Patient Care Pathways: Traumatic anterior shoulder instability” contributed to improved quality of care and provided a reduction in re-dislocations in the subsequent years [8,9]. The current clinical practice guidelines are intended to provide healthcare professionals with a standardized framework for the diagnosis and management of primary anterior shoulder dislocations.

## Methods

Initiated by the Dutch Society for Surgery (NVvH), and with support from the Knowledge Institute of the Federation of Medical Specialists, the current clinical practice guidelines were developed by a multidisciplinary committee consisting of orthopedic surgeons, radiologists, emergency physicians, physical therapists, an epidemiologist, and representatives from the Dutch Patient Federation [10]. The representatives were identified and invited by the professional associations, together with an advisory group comprising rehabilitation physicians, general practitioners, and anesthesiologists.

### Search and assessment strategy

Recommendations were formulated for: (i) diagnostic evaluation, (ii) reduction technique, (iii) pain management during reduction, (iv) immobilization, (v) physical therapy, (vi) risk factors for recurrent dislocation, and (vii) primary surgical shoulder stabilization. The recommendations were based on evidence synthesis from systematic literature reviews, randomized controlled trials (RCTs), and observational studies in Dutch or English, expert opinion, and on the Grading of Recommendations Assessment, Development, and Evaluation (GRADE) methodology. The search was based on clinical questions, formulated using the Population, Intervention, Control, and Outcome (PICO) framework (see Appendix). Medline, Embase, and Cochrane Database of Systematic Reviews were searched up to June 20, 2022. Medical information specialists guided the development of the search strategy (available upon request). Rayyan online software was used to remove duplicates and for the preliminary screening of studies [11]. 2 independent authors for each subtopic selected and reviewed the full-text version. In case of disagreement, a third author was consulted. The level of evidence was assessed using the GRADE approach (Table 1) [12]. The subtopic, primary diagnostics, relied on expert opinion, supported by a non-systematic literature review, since there was not 1 specific dilemma there on which to base a PICO (Table 2). A systematic review of the literature on pain management during reduction and physical therapy was conducted; however, no studies met the inclusion criteria.

**Table 1:** GRADE [12]

GRADE	Definition
High	We are very confident that the true effect is close to the estimate.
Moderate	We have moderate confidence in the effect estimate: The true effect is likely to be close to the effect estimate, but there is a possibility that it is substantially different.
Low	Our confidence in the estimate of the effect is limited: The true effect may be substantially different from the estimate.
Very low	We have very little confidence in the estimate of the effect: The true effect is likely to be substantially different from the estimate.

**Table 2:** Recommendations of the key questions in traumatic primary anterior shoulder dislocation

Recommendation / GRADE
**Diagnostic evaluation** In patients with a traumatic dislocation, standard radiographic imaging in a minimum of 2 orthogonal projections should preferably be obtained both prior to and following reduction. In cases where diagnostic uncertainty persists, CT is recommended. Ultrasonography should not be considered a substitute for conventional radiographs. GRADE: Expert opinion. No systematic literature search was conducted
**Reduction technique** Choose a reduction technique with which sufficient experience has been gained. A biomechanical reduction technique is advised as the first reduction technique. GRADE: Very low level of evidence
**Pain management during reduction** Discuss the different pain relief techniques with the patient. If reduction is unsuccessful, procedural sedation and analgesia (PSA) is the first-choice pain management technique. GRADE: Expert opinion. A comprehensive literature review was undertaken; however, no studies satisfied the established search criteria.
**Immobilization** Advise the patient to use a sling as needed for 1 week. GRADE: Very low level of evidence.
**Physical therapy** Discuss options for early restoration of rotator cuff function, coordination, and proprioception. If recovery is inadequate, refer to an orthopedic surgeon. In case of kinesiophobia, consider an interdisciplinary rehabilitation program GRADE: Expert opinion. A comprehensive literature review was undertaken; however, no studies satisfied the established search criteria.
**Risk factors for recurrent dislocation** Male sex and age < 40 years are risk factors for re-dislocation GRADE: A comprehensive literature review was performed. The studies could not be evaluated using the GRADE system.
**Indications for primary surgical shoulder stabilization** By shared decision, discuss surgical intervention in case of the following factors: age < 40 years, contact athlete, and significant bone loss. GRADE: Very low to moderate level of evidence

The revised Standards for Quality Improvement Reporting Excellence guidelines (SQuIRE 2.0) were adopted for the study reporting.

### Statistics

The 95% confidence interval (CI) is calculated as: point estimate ± critical value (z) × standard error of point estimate [13].

### Ethics, registration, data sharing plan, funding, and disclosures

The study was registered with the Dutch Federation of Medical Specialists. However, no public pre-registration was conducted, and no data sharing plan was formulated.

SDN and FB received financial compensation for the time spent on guideline creation. The OLVG (MPJvdB) receives fellowship support from Smith and Nephew.

No other author had conflicts of interest. Complete disclosure of interest forms according to ICMJE are available on the article page, doi: 10.2340/17453674.2026.46434

## Results

### Diagnostic evaluation

***Standard radiographic imaging in a minimum of 2 orthogonal projections should be obtained both prior to and following reduction. In the case of uncertainty, CT is recommended. Ultrasonography should not be considered a substitute for conventional radiographs.***

Patients arriving at the emergency department with a painful shoulder, suspected of primary dislocation, are enrolled in a diagnostic process to confirm or exclude a dislocation and any associated injuries. The patients present with a traumatic narrative, which is decisive for the diagnostic process. In the case of primary traumatic dislocations, though, pre- and post-reduction radiographs are recommended. Radiographs are primarily intended to confirm the diagnosis, determine the success of the reduction, and identify any additional (iatrogenic) fractures.

Conventional radiographs are the preferred method for diagnosing shoulder dislocation. In the majority of cases, radiographs are obtained in at least 2 projections, typically the anteroposterior view and scapular view [14]. The anteroposterior (AP) view provides a comprehensive assessment of shoulder abnormalities [15], and can detect up to 88% of all post-traumatic abnormalities [16]. Alternatively, the scapular Y-view (oriented towards the Y formation of the scapula, acromion, and coracoid) can facilitate visualization of potential anterior or posterior shoulder dislocation if the proximal humerus does not project over the glenoid. The advantages of the scapular Y-view include its relative ease of obtainment in cases of shoulder dislocation [15]. In cases where uncertainty persists, a CT scan can be performed to assess the extent of damage [17].

The use of ultrasound for diagnosing a dislocation was examined in a prospective cohort study on 65 patients with suspected shoulder dislocation and demonstrated a sensitivity and specificity of 100% (95% confidence interval 87–100) [18]. However, the study indicates that ultrasound is inadequate for diagnosing concomitant fractures, as 48% of all fractures were not detected.

Additional injuries, including Hill-Sachs lesions, glenoid lesions [19], and (iatrogenic) fractures of the greater/minor tuberosity, humeral head, and humeral neck [15], are frequently present in patients with shoulder dislocations. Post-reduction radiographs are crucial for identifying these fractures, as 15% of fractures detected in post-reduction imaging were not visible in pre-reduction radiographs [20]. Furthermore, the dislocations can be associated with neurological deficits and rotator cuff tears, while vascular damage remains a rare complication [15]. The management of these associated injuries is beyond the scope of this paper and is thus only briefly addressed.

### Reduction technique

***Choose a reduction technique with which sufficient experience has been gained. A biomechanical reduction technique is advised as the 1st reduction technique.***

Consider 3 different techniques of closed reduction to determine the most effective and least painful one [21]:

- Traction-based technique, where a longitudinal force is applied to the humerus to mitigate muscular spasm and facilitate relocation of the humeral head.
- Leverage techniques, where combined traction and lever forces are applied to manipulate the humeral head back into place in the glenoid fossa.
- Biomechanical or scapular reduction, intended to position the glenoid fossa in such a manner that it allows the humeral head to fall back into place. The patient is placed in prone position, followed by applying downward traction to the arm, which is flexed at 90 degrees. Subsequently, medial and upward pressure is exerted on the angulus inferior of the scapula.

The techniques are almost equally effective, with minimal complications and minor differences in pain experienced. Our opinion is that a biomechanical reduction technique should be recommended as the 1st reduction technique. The reduction is conducted, or overseen, by an emergency room physician, orthopedic surgeon, or trauma surgeon. If the initial attempt fails, a CT scan is employed to exclude complications, after which a subsequent reduction attempt is undertaken. In rare instances, the reduction must be performed in the operating theater or through an open reduction.

### Pain management during reduction

***Discuss the various pain-relief techniques with the patient. If reduction is unsuccessful, procedural sedation and analgesia (PSA) is the first-choice pain management technique.***

The ideal pain-relief method should be rapid-acting, highly efficacious, applicable in the acute setting, and not delay the reduction procedure. This can be achieved either without pain relief above the standard medication provided upon admission to the emergency department, moderately deep procedural sedation, or via an intra-articular lidocaine injection.

By applying moderately deep sedation, there is adequate pain relief to relax the surrounding muscles and successfully reposition the joint. Often, lidocaine injection alone is not sufficient, as illustrated by the limited patient satisfaction reported in a review comparing lidocaine injections with intravenous sedation [22]. Nitrous oxide was not included in these guidelines because of insufficient pain relief during reduction [23], and the intra-scalene block falls outside the standard availability in the acute setting. The medication routinely administered upon admission to the emergency department is encompassed by the Quality Standard for Inpatient Emergency Care [24].

### Immobilization

***Advise the patient to use a sling as needed for 1 week.***

In 1 RCT comparing wearing a sling as needed for 1 week with a prolonged immobilization period of 3 to 4 weeks, with a 2-year follow-up, no clinically relevant difference was found [19]. This means strict immobilization seems unnecessary, and the use of a sling as needed for 1 week alone can be recommended. Following the brief immobilization phase, physical therapy may commence, and activity can be gradually built up.

### Physical therapy

***Discuss options for early restoration of rotator cuff function, coordination, and proprioception. If recovery is inadequate, refer to an orthopedic surgeon. In the case of kinesiophobia, consider an interdisciplinary rehabilitation program.***

Initial guidance is provided in the emergency department, where patients typically receive a leaflet containing information and exercises designed to activate shoulder muscles and facilitate early, effective recovery. After a brief period of immobilization, rehabilitation may commence and, depending on individual physical and psychosocial factors, last for up to 6 months. After a traumatic primary anterior dislocation, a disruption of the capsulolabral complex could impair glenohumeral proprioception, lead to maladaptive movement patterns, and cause pain. Early activation and recruitment of the mechanical receptors, the rotator cuff, and kinetic chain muscles is therefore warranted [25], with primary focus on coordination and proprioception rather than strength [26].

Should non-surgical treatment prove unsuccessful, referral to an orthopedic surgeon experienced in shoulder dislocations for possible operative management should be considered. In patients exhibiting fear of re-dislocation, or kinesiophobia, affecting daily activities, work, or sport, an interdisciplinary rehabilitation program can be considered.

Shoulder dislocations carry a considerable risk of some level of neurological damage. In such instances, an assiduous physical therapy program is important to retain the range of motion until the muscle strength is regained and shoulder function is restored (12–45 weeks) [27].

### Risk factors for recurrent dislocation

***Male sex and young age are risk factors for re-dislocation.***

Intrinsic factors that demonstrated a significant association with shoulder instability, recurrent dislocation, or necessity for (secondary) surgery are age, sex, hyperlaxity, and greater tuberosity fractures. Being male and/or below 40 years is associated with a 3-fold and a 13-fold risk increase, respectively [28]. The recurrent dislocation occurrences vary across age groups (Table 3) [29]. A greater tuberosity fracture-dislocation, on the other hand, showed a negative association with shoulder instability, making recurrence less likely. Glenoid and Hill-Sachs lesions show an ambiguous association with recurrent instability following primary dislocations. Extrinsic risk factors of recurrent instability include collision sports, playing surface, and occupations involving work above shoulder height [28].

**Table 3:** Distribution of recurrent instability (N = 21,927) [29]

Age group	Women		Men	
	Dislocations, n	Recurrent dislocation (%)	Dislocations, n	Recurrent dislocation (%)
0–9	150	2.7	129	1.6
10–19	293	19	1,143	29
20–29	438	19	3,418	28
30–39	432	13	2,098	15
40–49	515	12	1,689	12
50–59	1,084	9.0	2,719	15
60–69	1,441	9.6	1,924	12
70–79	1,780	9.5	1,014	12
80–89	1,140	7.1	332	9.3
Total	7,273	10	13,683	19

### Primary surgical shoulder stabilization

***By shared decision, discuss options for surgical intervention in case of age < 40 years, contact athlete, and significant bone loss.***

Surgical stabilization of the shoulder comprises soft tissue repair, with and without remplissage or a more invasive osseous augmentation [30]. The recurrence of shoulder dislocation at a minimum of 2 years following soft tissue repair was reported in 1 systematic review and in 2 RCTs [31-33]. The first RCT handled the following inclusion criteria: (i) primary traumatic anterior dislocation, (ii) randomization ≤ 3 days after dislocation, (iii) type B2 (unidirectional without hyperlaxity) or B3 (unidirectional with hyperlaxity), and (iv) age 18–40 [32]. Patients received either arthroscopic Bankart repair or immobilization in 60° of external rotation and 30° of abduction, both within 3 days after dislocation. The second RCT included patients with (i) a radiologically confirmed primary anterior dislocation and (ii) age 18–25 years [33]. Patients received either 3 weeks of immobilization in internal rotation following dislocation or an arthroscopic Bankart repair within 15 days following dislocation. The results were pooled in a meta-analysis. 8.9% of patients in the surgical group experienced recurrence of shoulder dislocation compared with 49% in the nonoperative group. This resulted in a pooled relative risk of 0.2, favoring surgical treatment [31-33]. In the surgical group, 4.7% of patients had a positive apprehension test at 2-year follow-up, compared with 7.9% in the nonoperative group, although a nonsignificant difference [32]. The RCTs reported either no complications (apart from recurrent dislocations) or a single case of adhesive capsulitis at 3 months following Bankart repair [32,33]. The between-group differences in patient-reported outcome measures and ROM were not considered clinically relevant [32]. Return to sport at a minimum of 2 years follow-up was reported in 2 studies [32,33]. The pooled number of patients who returned to sport in the surgical group was 90%, compared with 62% in the nonoperative group.

A number needed to treat of 3 for dislocation and instability was found for patients without radiographic evidence of dislocation undergoing arthroscopic Bankart repair following primary dislocation [34].

Unfortunately, no studies on the outcome of the bone augmentation procedures were eligible for inclusion in the study.

## Discussion

We have established a standardized framework for the optimal diagnostic process and management of patients presenting to the emergency department with a traumatic primary anterior shoulder dislocation.

Guidelines are designed to offer a considered, impartial, evidence-based, accessible, and clear summary of how current health knowledge can be applied in practice, to enhance the quality of care when implemented as also demonstrated in subacromial pain syndrome [35-37]. The clinical practice guidelines suggested in our publication exhibit evident limitations. The recommendations of primary diagnostics were set up to rely exclusively on expert opinion and are a narrative overview of diagnostic options. On all other topics, systematic searches were conducted using thoroughly formulated clinical questions. Consequently, the outcomes were constrained by the search results, and for pain management during reduction and physical therapy, no studies were found eligible. The recommendations were therefore largely based on expert opinion. Reduction technique, immobilization, and primary surgical shoulder stabilization were based on a systematic review of the literature, but the evidence level for the included studies ranged from very low to moderate. An additional limitation of the study was the exclusion of patients with firsthand experience of a primary traumatic shoulder dislocation.

The use of pre- and post-reduction radiographs is crucial for accurately diagnosing concomitant fractures. An iatrogenic fracture is a complication of the closed reduction process, mostly seen in patients older than 40 years, and predominantly in women with a concomitant fracture of the greater tuberosity. The choice of treatment should be individualized depending on age, fracture severity, overall medical condition, and functional demands. Efficient and timely management is crucial for the functional outcome [4]. Given the complexity and demanding nature of these injuries, it is imperative to engage in shared decision-making. It is recommended that these procedures be conducted promptly, once a rested and qualified team is available.

Besides the concomitant fractures, rotator cuff lesions were found in more than half of the dislocated shoulders [38]. In a recent investigation of symptomatic shoulders in individuals over 40 years old, 98% were found to have a rotator cuff lesion [39]. Careful anamnesis of pre-existing symptoms vs acute onset of pain and weakness is therefore important for the choice of treatment.

Although only 1 complication, adhesive capsulitis, was identified following surgery [33], this single finding may not provide a comprehensive view of the potential adverse events. Complications can occur at a frequency of 1–14%, including a variety of issues such as hardware problems, (iatrogenic) fracture, hematoma, infection, postoperative stiffness, nerve injury, stitch abscess, adhesive capsulitis, non-union, graft osteolysis/displacement, early arthritis, bursitis/tendinitis, superficial vein thrombosis, and complex regional pain syndrome (CRPS) in different types of stabilizing surgical procedures [40]. Therefore, early recognition, thorough diagnostics, and, if needed, referral to a specialist, is imperative to deal with surgery-related complications.

The clinical practice guidelines demonstrate strengths as well. To ensure their comprehensiveness and minimize bias, the current clinical practice guidelines were formulated by a diverse group of specialized healthcare professionals in collaboration with members of the Dutch Patient Federation. The recommendations were substantiated by evidence wherever feasible; otherwise, they relied on the clinical experience of the professionals. For clinical applicability, aspects such as potential harm and organizational factors were considered.

An urgent need remains for high-quality research to substantiate future guidelines and to continuously evaluate the outcomes of their implementation.

### Conclusion

Our clinical practice guidelines emphasize the importance of timely, efficient, and safe management in the emergency department, where pre- and post-reduction diagnostics are crucial for safe reduction and appropriate management. Following the acute phase, identifying risk factors for recurrent instability is important, as this forms the foundation for shared decision-making regarding whether to pursue conservative or surgical treatment of persisting instability complaints. This treatment choice is vital for the long-term outcomes and the likelihood of patients returning to their previous levels of activity.

## Notes

Conceptualization: all authors. Methodology: all authors. Data curation: all authors. Formal analysis: all authors. Writing—original draft: all authors. Writing—review and editing: all authors. Approval of final manuscript: all authors.

Handling co-editors: Cecilia Rogmark and Robin Christensen

*Acta* thanks Eirik Aunan and Carl Ekholm for help with peer review of this manuscript.
